# Peripheral Nerve Stimulation for Treating Acute Pain Following Traumatic Fracture: A Case Report of Rapid-Onset Analgesia Without Motor Blockade

**DOI:** 10.7759/cureus.62142

**Published:** 2024-06-11

**Authors:** Ammar Siddiqui, Nitin Sekhri, Irim Salik, Fang Yu, Jeff L Xu

**Affiliations:** 1 Department of Anesthesiology, Westchester Medical Center/New York Medical College, Valhalla, USA; 2 Department of Neurology, Westchester Medical Center/New York Medical College, Valhalla, USA

**Keywords:** rapid-onset analgesia, motor blockade sparing, nerve blockade, traumatic fracture, peripheral nerve stimulation

## Abstract

Analgesia following acute traumatic fracture remains a clinical challenge. Pain relief via peripheral nerve stimulation (PNS) is a promising treatment modality due to its opioid-sparing effects and rapid, reversible sensory blockade without motor blockade. We present the case of a patient who suffered a traumatic tibial plateau fracture. A popliteal sciatic PNS device was placed on postoperative day 1 following inadequate pain control. The patient reported marked pain relief, a significant reduction in morphine milligram equivalent (MME) utilization, and improved early functional recovery. The PNS lead was removed at the patient’s 2-month follow-up visit without any adverse events.

## Introduction

Acute fracture pain, resulting from skin, substance tissue, periosteal, muscular, and nerve damage, can be challenging to treat. While the coordinated use of acutely fractured bones is necessary for patients to preserve long-term mobility, poorly controlled acute pain can complicate early ambulation and increase the risk of chronic pain [1,2]. Neural mechanisms contribute significantly to the transduction and transmission of fracture pain. Distortion of mechanosensitive nerve fibers following fracture leads to intraosseous pressure changes along with peripheral neuroinflammation, causing sensitization to mechanical stimulation [3].

Peripheral nerve stimulation (PNS) has emerged as a promising treatment for both nociceptive and neuropathic pain following percutaneous device implantation. PNS has been utilized successfully for a variety of neuropathic pain conditions, such as phantom limb pain, acute postoperative pain, complex regional pain syndrome, and peripheral neuropathy, and somatic conditions, such as osteoarthritis and hemiplegic shoulders [2,4]. Given the high neural contribution to fracture pain, we utilized PNS to treat acute traumatic fracture pain, while sparing motor function. This manuscript adheres to the applicable Enhancing the Quality and Transparency of health Research (EQUATOR) guidelines.

## Case presentation

**Figure 1:**
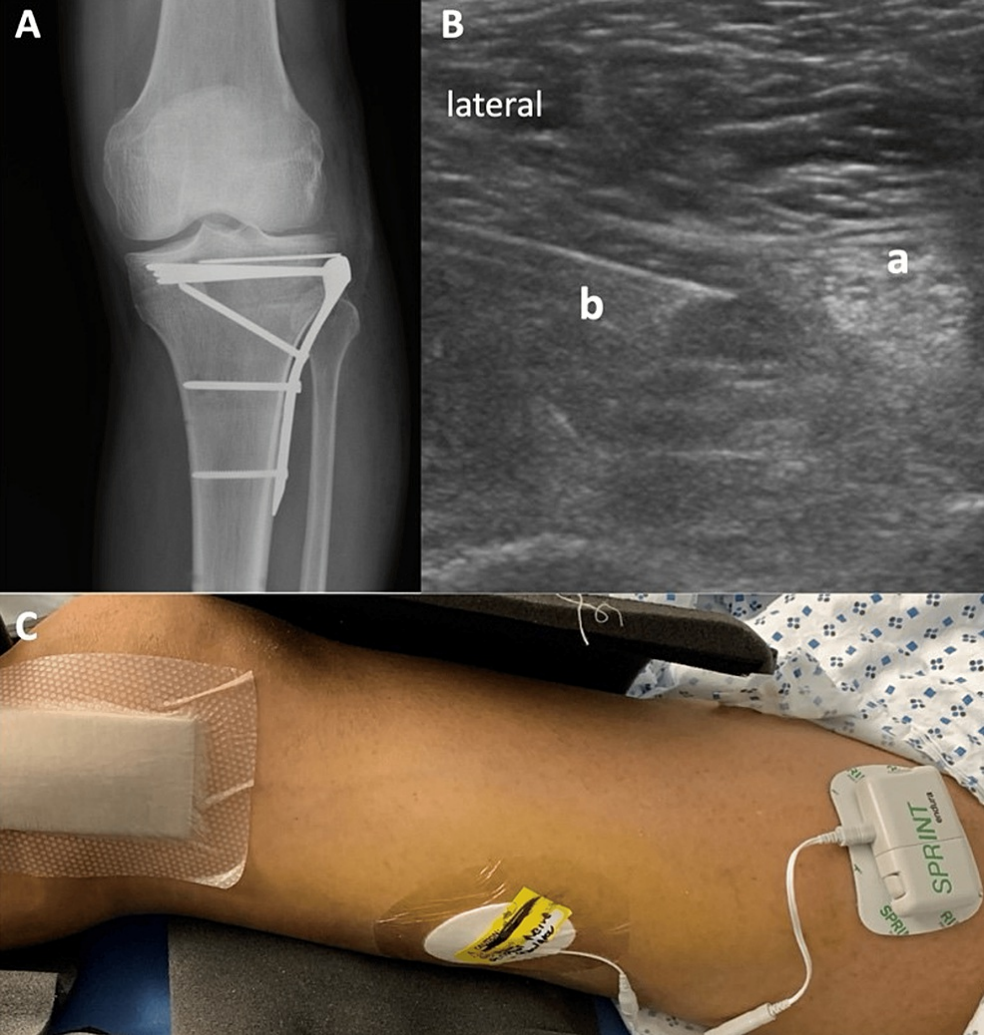
Images of peripheral nerve stimulation (PNS) placement Figure 1: (A) Postoperative X-ray imaging following open reduction and internal fixation of the tibial plateau fracture, (B) Ultrasound-guided needle/electrode placement for PNS placement, and (C) the PNS system a: Sciatic Nerve, b: Needle

A 52-year-old, 68 kg male patient presented with a left-sided tibial plateau fracture after being struck by an automobile as a pedestrian. The patient underwent open reduction and internal fixation of the fracture on the second day following traumatic injury. (Figure 1A). Postoperative pain remained uncontrolled despite multimodal analgesia. A regional nerve block was declined by the surgical team due to the high risk of compartment syndrome given the patient’s complex fracture presentation and significant postoperative swelling. On postoperative day 1, a 60-day non-surgical temporary PNS system (SPRINT^@^, Cleveland, OH, USA) was inserted via ultrasound guidance by the acute pain team. Ultrasound with a high-frequency (10-12 MHz) linear probe (Philips, Bothell, WA, USA) was used to identify the popliteal sciatic nerve 4-cm cephalad to the popliteal fossa. After local infiltration with 5 ml of 1% lidocaine, a 17-gauge hollow bore introducer needle and a 19-gauge stimulating probe were inserted via the lateral approach towards the sciatic nerve (Figure 1B). A 1 cm long stimulating unipolar microlead was placed perineurally until a pleasant paresthesia was elicited by the patient at a sensory stimulation level of 100 Hz. As the probe passed close to the sciatic nerve, the patient experienced stimulation at 20 mA, with a pulse duration range from 20 to 80 microseconds. Following connection to an external stimulator (Figure 1C), the device was programmed to generate sensations of comfort in the area of pain.

**Figure 2:**
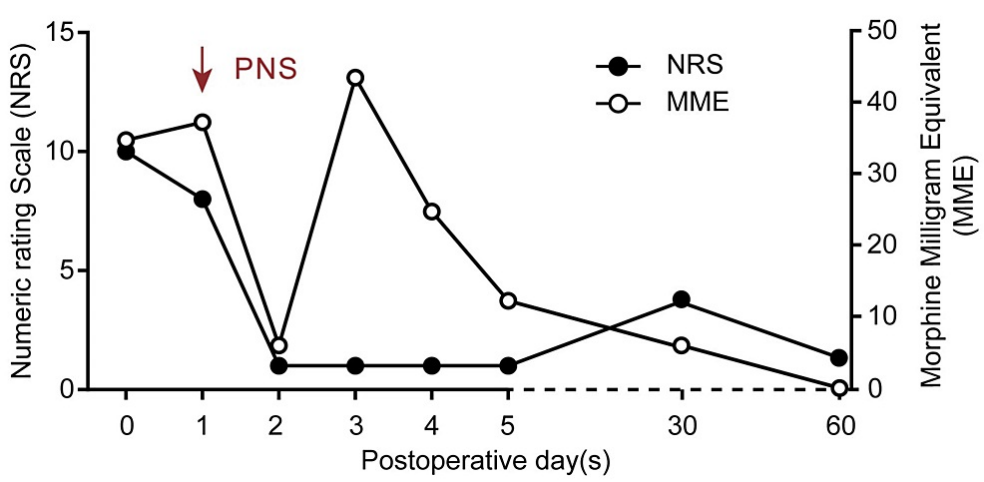
Post-PNS placement pain score and opioid usage Figure 2: Post PNS placement Numeric Rating Scale (NRS) scores and oral Morphine Milligram Equivalent (MME) over time.

The patient reported an 80% reduction in pain intensity immediately after PNS implantation. Opioid requirements and reported pain intensity decreased dramatically during the first day after PNS implantation, though the pain and opioid requirements did increase once the patient started physical therapy on POD 3 (Figure 2). A knowledge gap was identified in that the patient was turning off his PNS during therapy sessions on POD 4. Once the patient was encouraged to keep his PNS on during therapy, opioid consumption subsequently decreased on POD 5. The patient was discharged home on postoperative day 5 with his PNS in place and followed up as an outpatient in the pain clinic, where he reported excellent pain relief without interference in functional recovery or motor weakness. During this period he was only taking acetaminophen 650 mg every 4 to 6 hours as needed and 5 mg of oxycodone daily. The PNS lead was removed uneventfully at the patient’s 2-month follow-up. No side effects were reported by the patient.

## Discussion

In conjunction with the current literature, our case report adds evidence of percutaneous PNS as an important analgesic adjunct following acute trauma, leading to reduced opioid utilization and improved function. Uncontrolled acute postoperative pain following trauma may not only lead to years of subsequent chronic pain, but reliance on opioids as the primary means of analgesia has resulted in soaring rates of opioid abuse, dependence, and addiction [5]. Oelreich et al found that traumatic injury despite surgical intervention is associated with chronic opioid use and increased mortality risk 6-18 months after injury [6]. Mauck et al also found new persistent opioid use was observed in 20% of patients hospitalized after orthopedic trauma [7]. Trauma patients in particular might be at elevated risk of opioid use depending on the nature of the injury and coexisting comorbidities, particularly in those with chronic fracture pain.

Hospital-based anesthetic nerve blockade usually lasts less than 24 hours, while continuous catheters are not utilized for longer than 5-7 days due to an increased risk of infection and migration [8,9]. In addition, the afferent blockade created by local anesthetics is normally accompanied by a motor blockade that can limit mobility and participation in early physical therapy. Tibial shaft fractures, as seen in our patient, are recognized as one of the highest-risk injuries for acute compartment syndrome [10]. There is a possibility that regional blockade or catheter placement may mask the pain of compartment syndrome, and their use is limited in some injuries [11]. Our regional anesthesia team sought to reduce persistent pain and opioid use in selected postoperative trauma patients by implementing percutaneous PNS as an adjunct to the aforementioned analgesic options.

PNS utilizes a percutaneously implanted electrode along a peripheral nerve, causing a nociceptive response. With relatively few adverse events and a lead infection risk of less than 1 per 30,000 indwelling days, PNS seems to have a relatively strong safety profile [12]. In the literature, PNS has been applied to the military population with traumatic orthopedic injuries, in patients with a total knee replacement, and in postamputation pain, finding that its application reduces postoperative pain and opioid consumption, and improves functional recovery following orthopedic trauma [12, 13]. Additionally, PNS has previously been studied to investigate its utility in chronic pain relief for amputees, showing improved function and an 82% reduction in pain interference with daily activities [14]. In a pilot study conducted by Ilfeld et al, PNS was compared to “sham” or placebo electric stimulation in postoperative patients, finding that the intervention group had reduced pain scores and opioid requirements for at least a week after ambulatory orthopedic surgery [15].

While PNS appears to show some promise for both acute and chronic pain from fractures, it does have several limitations. Primarily, it is a costly apparatus with an estimated cost of $4900 for the device alone. In addition, it requires appropriate settings for device implantation and supervised management by a PNS controller. Although low risk, there remains the possibility of infection with implanted electrodes, as well as the risk of lead fracture during physical therapy. Of note, the device is MRI-incompatible.

## Conclusions

PNS represents a promising analgesic option in patients suffering from traumatic fractures without inciting motor blockade. Randomized controlled trials in patients with orthopedic trauma are integral to elucidate the risks and benefits of percutaneous PNS in the postoperative period. Although our case yields optimistic findings, further research is warranted before validating PNS as a standard modality in this patient population. 
